# Argininemia and plasma arginine bioavailability – predictive factors of mortality in the severe trauma patients?

**DOI:** 10.1186/s12986-016-0118-6

**Published:** 2016-08-31

**Authors:** Beatriz P. Costa, Paulo Martins, Carla Veríssimo, Marta Simões, Marisa Tomé, Manuela Grazina, Jorge Pimentel, Francisco Castro-Sousa

**Affiliations:** 1 “A” Surgical Department, Hospitais da Universidade de Coimbra, Centro Hospitalar e Universitário de Coimbra, Praceta Prof. Mota Pinto, 3000-075 Coimbra, Portugal; 2 Faculty of Medicine, University of Coimbra, Coimbra, Portugal; 3 Intensive Medicine Department, Centro Hospitalar e Universitário de Coimbra, Coimbra, Portugal; 4 Genetic Biochemistry Department, Center for Neurosciences and Cellular Biology of Coimbra University, Faculty of Medicine, University of Coimbra, Coimbra, Portugal

**Keywords:** Arginine, Plasma arginine bioavailability, Trauma, Mortality, Critically ill patients

## Abstract

**Background:**

Arginine is an amino acid determinant in the metabolic, immune and reparative responses to severe trauma. The present study aims to determine argininemia and plasma arginine bioavailability (PAB) in critical trauma patients and to analyze its correlation with prognosis.

**Methods:**

A prospective study of 23 critical trauma patients was undertaken. Aminoacidemias were determined, by ion exchange chromatography, at admission and in the first and third days and compared with those of 11 healthy individuals. PAB was calculated. Severity indexes and outcome parameters were recorded.

**Results:**

Values of argininemia, citrullinemia and ornithinemia at the admission were significantly lower than those of the controls (arginine: 41.2 ± 20.6 versus 56.1 ± 11.9 μmol/L, *P* = 0.034). Hipoargininemia (<60 μmol/L) prevalence was 82.6 %. Mean PAB was 62.4 ± 25.6 %. Argininemia < 26 μmol/L constituted a significant predictive factor of in-hospital mortality [*n* = 4 (17.4 %); 75 versus 15.8 %, *P* = 0.04; *odds* ratio = 4.7; accuracy = 87 %] and lower actuarial survival (63.5 ± 43.9 versus 256.1 ± 33.3 days, *P* = 0.031). PAB <42 % [*n* = 6 (26.1 %)] was associated with higher lactacidemia levels (*P* = 0.033), higher in-hospital mortality (66.7 versus 11.8 %, *P* = 0.021; *odds* ratio = 5.7, accuracy = 82.6 %) and lower actuarial survival (87.2 ± 37.5 versus 261.4 ± 34.7 days, *n.s.*). Probability of in-hospital mortality was inversely and significantly related with PAB [61.8 ± 8.8 % (95 % CI 50.8–72.7) when PAB <41 % and 2.8 ± 1.9 % (95 % CI 1.9–8.3) when PAB > 81 %, *P* = 0.0001]. Charlson’s index ≥1, APACHE II ≥19.5, SOFA ≥7.5, and glutaminemia < 320 μmol/L were also predictive factors of actuarial survival.

**Conclusions:**

Those results confirm the high prevalence of arginine depletion in severe trauma patients and the relevance of argininemia and PAB as predictive factors of mortality in this context.

## Background

Arginine is a conditionally essential amino acid involved in protein synthesis; ureagenesis and ammonia detoxification; nitric oxide metabolism; production of proline (used for collagen synthesis and tissue repair), polyamines (primary regulators of cellular growth and proliferation), creatine and agmatin; and hormonal secretion (including growth hormone, insulin and prolactin) [1–4]. Arginine participates in the modulation of the immune function (including T-lymphocytes proliferation and activation), inflammatory response, tissue perfusion, wound healing and airway tonus control [1–3].

Circulating arginine derives from protein turnover, *de novo* endogenous synthesis (in the kidney from gut-produced citrulline via the urea cycle) and dietary protein sources [4]. Arginine is metabolized predominantly by two competing pathways, namely nitric oxide synthases (NOSs) and arginases (I and II) as part of the urea cycle. Arginases transform arginine into ornithine (precursor of proline and polyamines) and urea, whereas NOSs convert arginine into nitric oxide and citrulline. Arginase I is found in the cytosol of hepatocytes and leukocytes, while arginase II is a mitochondrial enzyme present in the macrophages and numerous other cells [4, 5]. Three isoforms of NOS are relevant: NOS 1 (neuronal) and NOS 3 (endothelial) that are constitutive enzymes; and NOS 2 (inducible) that is markedly induced during inflammation. Both arginase I and inducible NOS (iNOS) are inducible enzymes in myeloid cells, with arginase I being induced by T-helper 2 (Th2) cytokines and iNOS by T-helper 1 (Th1) cytokines [4–6]. Preponderant type of reaction is influenced by the nature of injury [7], namely Th2 response in trauma and *major* surgery and Th1 response in sepsis.

Systemic arginine availability has been estimated by calculation of the ratios of arginine to its enzymatic products (citrulline and ornithine) and arginine to its endogenous metabolic inhibitors (asymmetric and symmetric dimethylarginines) [8]. Plasma arginine bioavailability (PAB) is defined by the argininemia-to-(citrullinemia plus ornithinemia) quotient [9, 10]. PAB constitutes an indirect measure of the arginine endogenous synthesis and of the arginase activity; and, also, a reflex of the oxide nitric production [9], which is recognized as an important signaling agent with vasodilatation, cytotoxicity and neurotransmission effects [2, 11–13]. PAB is considered, in several contexts, a bioindicator of arginine metabolism deregulation more accurate than isolated argininemia [9, 10].

Arginine is determinant in the metabolic, immune and reparative responses to severe trauma [14], which constitutes the leading cause of mortality in the first four decades of life [15, 16]. Arginine depletion is frequent in severe trauma patients and has a potential relevant impact in the prognosis [14, 15].

Present study aims to determine the profile of plasma concentrations of arginine, citrulline and ornithine in critical trauma patients; to calculate plasma arginine bioavailability; and to analyze their correlation with the severity indexes and the clinical outcome.

## Methods

A single-center observational prospective study of adult critical trauma patients non-electively admitted in the Intensive Care Unit (ICU) of Hospitais da Universidade de Coimbra, Centro Hospitalar e Universitário de Coimbra, Coimbra, Portugal was undertaken between October 2013 and April 2014. Included patients fulfilled the Intensive Care Society definition of critically illness [17] and were expected to require an ICU length of stay of at least three days. Exclusion criteria included pregnancy, lactation, acquired immunodeficiency syndrome, renal insufficiency (creatininemia ≥ 2 mg/dL), acute liver failure (defined according the criteria previously described by O’Grady JG et al. [18] and Moreau R et al. [19]) and amino acid metabolism diseases.

Study was approved by the institution’s ethics committee (Centro Hospitalar e Universitário de Coimbra, Coimbra, Portugal; Official Letter n° CHUC00115) and was performed following the principles established by the Helsinki’s declaration [20].

Patients’ demographic characteristics were acquired, including age and gender. Type of admission was characterized as primary or non-primary (after previous initial care on peripheral hospitals). Severity indexes were registered at the moment of admission, including Acute Physiology and Chronic Health Evaluation II (APACHE II) score [21], Simplified Acute Physiology Score II (SAPS II) [22], Sequential Organ Failure Assessment (SOFA) score [23], Abbreviated Injury Scale [24], Injury Severity Score [25], Revised Trauma Score [26] and Shock Index [27]. In addition, comorbidities were described with the Charlson’s index [28]. Invasive ventilation, erythrocytes transfusions, amines perfusion, renal replacement therapy, surgical procedures and nutritional support were recorded, as well as, glutamine exogenous supplementation. Standard formulas were used in enteral nutrition support; intravenous administration of glutamine was prescribed in patients submitted to parenteral nutrition, at the dose of 0.2–0.4 mg/kg/day.

Evaluation was performed at the moment of admission in the ICU, at the first and the third days, with determination of amino acid plasma levels (arginine, citrulline, ornithine, glutamine, alanine, proline, glutamic acid, leucine and isoleucine) and regular laboratory tests (including blood gases analysis; lactacidemia; serum biochemistry with hepatobiliary enzymes, ionograme, creatinine, albumin, lactate dehydrogenase, creatinephosphokinase and C-reactive protein; hemograme; caolin-cefalin and prothombin times).

Plasma concentrations of amino acids were studied by ion exchange chromatography in a high pressure system (Biochrom 30 analyzer). Plasma was extracted from blood sampled in ethilenediaminotetraacetic acid, by centrifugation at 4000 g, during 10 min, and reserved at 4 °C; samples were prepared with 12 % ditiotreitol, five to 10 min, deproteinized with sulfosalicilic acid, 60 min at room temperature and, after separation of the sediment by centrifugation, were filtered and preserved at −20 °C for subsequent analysis.

Plasma arginine bioavailability (PAB) was calculated in accordance to the formula: argininemia:(citrullinemia + ornithinemia) [9, 12] and expressed in percentage. Arginase activity was estimated by the argininemia-to-ornithinemia ratio [8].

Amino acid plasma concentrations of critical trauma patients were compared with those of a historical control group of eleven fasting healthy individuals [29].

Primary end-points included in-hospital mortality rate and actuarial survival. Secondary end-points were health care-associated infections rate [30], duration of invasive ventilation support, hospital and ICU lengths of stay and performance *status* at the moment of the last observation (characterized by the Karnofsky index [31]). Health care-associated infections were defined according the surveillance definition in the acute care setting of the National Healthcare Safety Network (NSHN), Centers for Disease Control and Prevention (CDC), Atlanta, GA, USA [30].

Statistical analysis was performed with SPSS Software version 18.0 for Windows (SPSS Inc., Chicago, IL, USA). Qui-square, Student’s *t,* paired Student’s *t*, Kaplan Meier and log rank tests, multivariable logistic regression, Cox’s regression, Pearson’s correlations and Receiver Operating Characteristic (ROC) curves were used. Level of significance was considered *P* < 0.05. Data were presented as n (%) or mean ± standard deviation (SD).

## Results

Twenty-three critical trauma patients were studied and presented the characteristics described in Table 1. Determination of plasma amino acid profile was accomplished in all patients at the ICU admission; in 18 both at the admission and the first day; only 12 patients completed the three moments of evaluation.

**Table 1:** Critical trauma patient’s characteristics (*n* = 23)

	*n* (%) or mean ± SD
Male gender	18 (78.3)
Age (years-old)	48.8 ± 17.8 (21–82)
Charlson’s comorbidity index	0.78 ± 1.98 (0–8)
Primary admission	18 (78.3)
APACHE II	19.4 ± 5.5 (10–32)
SAPS II	41.3 ± 12.2 (20–78)
SOFA	6.9 ± 3.2 (2–10)
Shock index	0.82 ± 0.25 (0.31–1.4)
Abbreviated Injury Scale	15.4 ± 4.9 (10–25)
Injury Severity Score	47.9 ± 18.5 (27–75)
Revised Trauma Score	5.9 ± 1.3 (3.6–7.6)
Invasive ventilation	23 (100)
Renal replacement therapy	0 (0)
Amines perfusion	16 (69.6)
Erythrocytes transfusion	14 (60.9)
Nutritional support	
Enteral/Parenteral	23 (100)/1 (4.3)
Glutamine supplementation^a^	1 (4.3)
Surgical procedures^b^	14 (65.2)
Mortality rate	
ICU/Hospital/Global	4 (17.4)/6 (26.1)/10 (43.5)
Health care-associated infections rate	20 (87)
Ventilation support (days)	12.7 ± 7.8 (2–27)
Length of stay (days)	
ICU/Hospital (days)	13.9 ± 9.1 (3–52)/29.4 ± 21.9 (5–95)
Follow-up (months)	7.4 ± 3.1 (2.3–12.2)
Actuarial survival (days)	229.2 ± 32.9 (95 % CI 164.7–293.8)
Karnofsky’s index	69 ± 17.3 (40–90)

Data presented as number (%) or mean ± standard deviation

*APACHE II* acute physiology and chronic health evaluation II, *ICU* intensive care unit, *SAPS II* simplified acute physiology score II, *SD* standard deviation, *SOFA* sequential organ failure assessment

^a^Glutamine was administered after the third day in the intensive care unit

^b^Operations before intensive care unit admission (*n* = 4); operations after the third day in intensive care unit (*n* = 5)

**Fig. 1:**
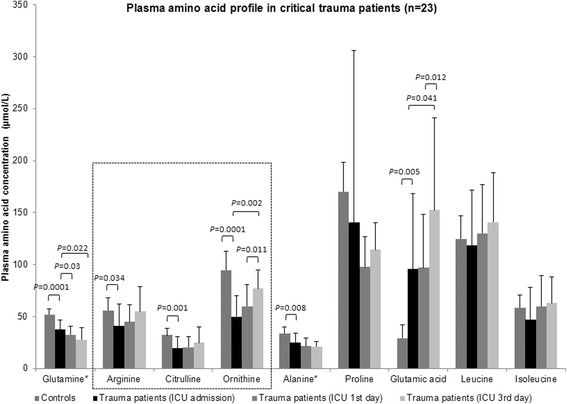
Mean aminoacidemia levels in critical trauma patients (*n* = 23) at the moment of the admission in the intensive care unit (ICU) and at the first and the third days; and in control healthy individuals (*n* = 11). Comparisons were performed with Student’s-*t* test (with control group) and paired Student’s-*t* test (between the ICU moments of evaluation). * Plasma levels × 10^−1^. Controls corresponded to a historical cohort of healthy individuals [29]

**Fig. 2:**
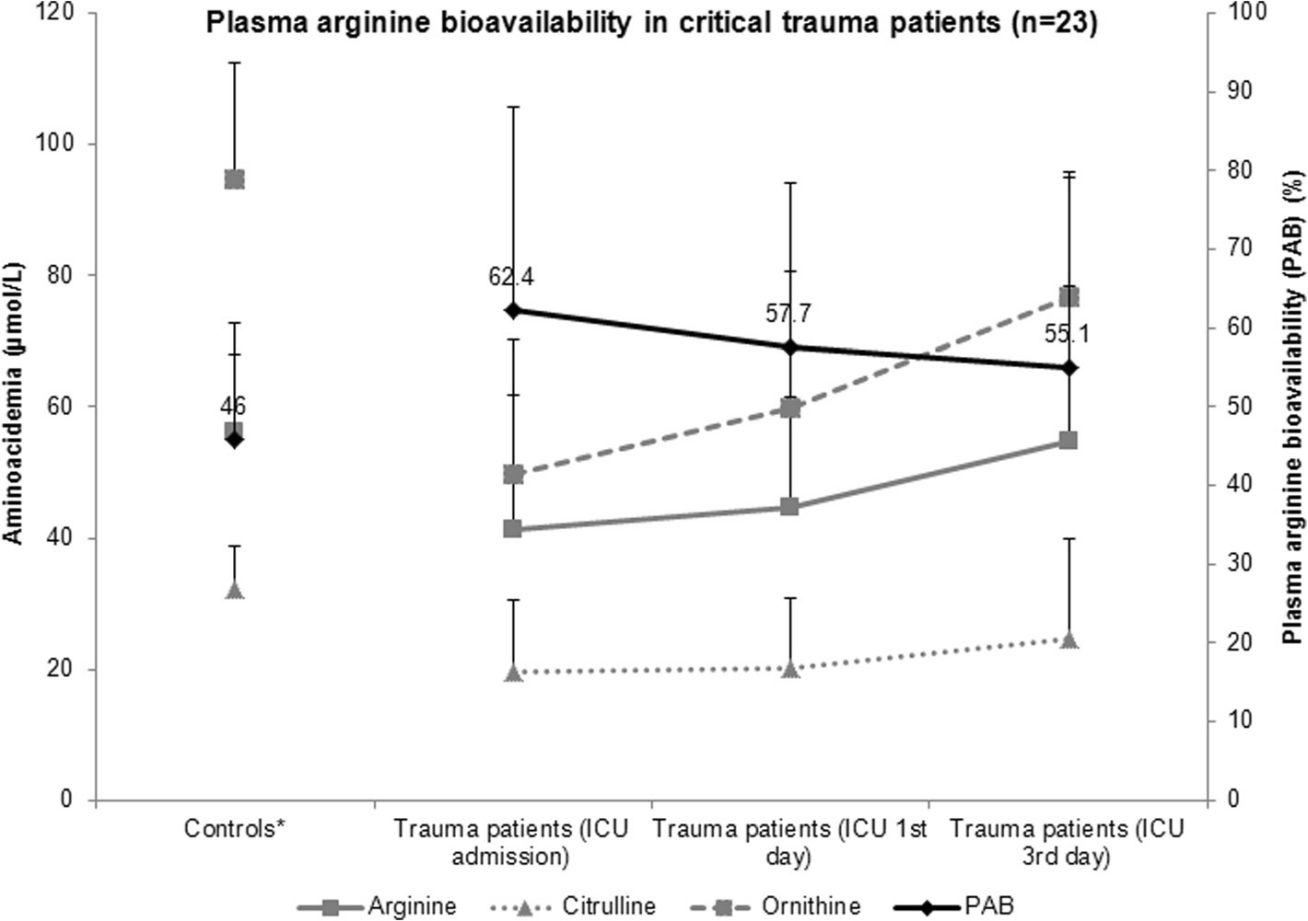
Mean plasma arginine bioavailability (PAB), argininemia, citrullinemia and ornithinemia in critical trauma patients (*n* = 23) at the moment of the admission in the intensive care unit (ICU) and at the first and the third days; and in control healthy individuals (*n* = 11). Controls corresponded to a historical cohort of healthy individuals [29]

At the moment of admission in ICU, critical trauma patients demonstrated lower mean values of argininemia, citrullinemia and ornithinemia than fasting historical control individuals (argininemia: 41.2 ± 20.6 versus 56.1 ± 11.9 μmol/L, *P* = 0.034; citrulline: 19.5 ± 11.1 versus 32.2 ± 6.6 μmol/L, *P* = 0.001; ornithine: 49.6 ± 20.6 versus 94.6 ± 17.9 μmol/L, *P* = 0.0001); they also exhibited lower mean levels of glutaminemia and alaninemia and higher mean glutamic acid plasma concentration (Fig. 1). Hipoargininemia (argininemia inferior to 60 μmol/L) prevalence at the ICU admission was high (82.6 %). Initial mean plasma arginine bioavailability (PAB) in severe trauma patients was 62.4 ± 25.6 %, not significantly different from that of control individuals (46 ± 14.7 %) (Fig. 2). Argininemia-to-ornithinemia ratio in trauma patients at the ICU admission was higher than in control subjects (89.2 ± 36.2 versus 61.9 ± 20.5 %, *P* = 0.009).

During the first three days of ICU stay, a decrease of glutaminemia and an increase of ornithinemia and glutamic acid plasma levels were observed; no significant differences were verified in the remaining amino acid concentrations (including argininemia and citrullinemia), PAB or argininemia-to-ornithinemia ratio between the three moments of evaluation.

At the moment of admission, argininemia and PAB did not correlate significantly with patients’ age, Charlson’s index and severity scores, including APACHE II, SAPS II, SOFA, Abbreviated Injury Scale, Injury Severity Score, Revised Trauma Score and Shock Index; furthermore, no significant differences were observed in those parameters according gender, type of admission or submission to previous surgical procedures. Argininemia at the moment of admission correlated significantly with glutaminemia [Pearson’s correlation coefficient (*r*) = 69.5 %, *P* = 0.0001], ornithinemia (*r* = 55.6 %, *P* = 0.006), C-reactive protein (*r* = 71.1 %; *P* = 0.006) and albuminemia (*r* = 70.1 %; *P* = 0.008).

Argininemia values lower than 26 μmol/L at the admission were significantly associated with higher in-hospital mortality rate, shorter duration of invasive ventilation and ICU length of stay and lower mean actuarial survival (Table 2). PAB levels inferior to 42 % at the admission were significantly associated with higher in-hospital mortality rate. Initial argininemia and PAB values were not significantly related with the development of nosocomial infections.

**Table 2:** Clinical outcome of critical trauma patients (*n* = 23) according to argininemia and plasma arginine bioavailability at the ICU admission

	Argininemia <26 μmol/L vs ≥26 μmol/L	*P* ^a^	Plasma arginine bioavailability <42 % vs ≥42 %	*P* ^a^
In-hospital mortality (%)	75 vs 15.8	0.04	66.7 vs 11.8	0.021
Health care-associated infections rate (%)	75 vs 89.5	n.s.	83.3 vs 88.2	n.s.
Invasive ventilation period (days)	6.3 ± 3.5 vs 14.1 ± 7.8	0.01	8.5 ± 6.7 vs 14.2 ± 7.7	n.s.
ICU length of stay (days)	7 ± 3.6 vs 15.4 ± 9.3	0.01	9.5 ± 6.7 vs 15.5 ± 9.5	n.s.
Hospital length of stay (days)	15.3 ± 9.3 vs 32.4 ± 22.8	n.s.	21.8 ± 17.8 vs 32.1 ± 23.1	n.s.
Mean actuarial survival (days)	63.5 ± 43.9 vs 256.1 ± 33.3	0.031	87.2 ± 37.5 vs 261.4 ± 34.7	n.s.
Performance status (Karnofsky’s index)	50 ± 0 vs 71.1 ± 16.9	n.s.	65 ± 21.2 vs 70 ± 17.7	n.s.

Data presented as number (%) or mean ± standard deviation

*ICU* intensive care unit, vs versus

^a^Qui-square, Student’s-*t*, Kaplan Meier and *log rank* tests

In univariate analysis, Charlson’s index superior or equal to one, argininemia lower than 26 μmol/L and PAB inferior to 42 % were risk factors of in-hospital mortality; only Charlson’s index was significant in multivariate analysis (*P* = 0.045) (Table 3). Cases of in-hospital mortality demonstrated lower mean plasma concentrations of arginine, glutamine and alanine and higher mean levels of proline and glutamic acid, however without reaching statistically significant differences.

**Table 3:** Univariate analysis of in-hospital mortality and actuarial survival in critical trauma patients (*n* = 23)

	(%)	In-hospital mortality (%)	*P* ^a^	Mean survival (days)	*P* ^b^
Male gender	(78.3)	27.8 vs 20	n.s.	218.2 ± 36.4 vs 243.6 ± 66.5	n.s.
Age ≥ 60 years-old	(30.4)	42.9 vs 18.8	n.s.	103.7 ± 30.7 vs 265.5 ± 37.5	n.s.
Charlson’s Index ≥ 1	(17.4)	75 vs 15.8	0.04	57 ± 34.3 vs 266.2 ± 33.8	0.005
APACHE II ≥ 19.5	(50)	45.5 vs 9.1	n.s.	152 ± 43.5 vs 310.7 ± 35.4	0.031
SAPS II ≥ 39.5	(54.6)	41.7 vs 10	n.s.	167.3 ± 42.4 vs 305 ± 38.6	n.s.
SOFA ≥ 7.5	(53.9)	42.9 vs 0	n.s.		0.039
Injury Severity Score < 65	(73.9)	35.3 vs 0	n.s.	192.2 ± 37.6 vs 315.7 ± 45	n.s.
Revised Trauma Score ≥ 4.8	(78.3)	33.3 vs 0	n.s.		n.s.
Shock index ≥ 0.75	(54.6)	41.7 vs 10	n.s.	196.5 ± 48.9 vs 273.3 ± 39.3	n.s.
Primary admission (yes vs no)	(21.7)	40 vs 22.2	n.s.	173.6 ± 70.7 vs 237.2 ± 35.1	n.s.
Lactacidemia ≥ 1.25 mmol/L	(63.6)	35.7 vs 12.5	n.s.	207.6 ± 42.9 vs 275.3 ± 48	n.s.
Arginine bioavailability < 42 %	(26.1)	66.7 vs 11.8	0.021	87.2 ± 37.5 vs 261.4 ± 34.7	n.s.
Argininemia < 26 μmol/L	(17.4)	75 vs 15.8	0.04	63.5 ± 43.9 vs 256.1 ± 33.3	0.031
Glutaminemia < 320 μmol/L	(21.7)	60 vs 16.7	n.s.	70.6 ± 34.1 vs 264.8 ± 34.2	0.02
Citrullinemia ≥ 22 μmol/L	(26.1)	50 vs 17.6	n.s.	151.5 ± 52.8 vs 241.2 ± 36.6	n.s.
Ornithinemia ≥ 72 μmol/L	(17.4)	50 vs 21.1	n.s.	182.5 ± 76.7 vs 235.7 ± 35.6	n.s.
Arg-to-Orn ratio < 77 %	(47.8)	45.5 vs 8.3	n.s.	172.1 ± 45.6 vs 268.7 ± 40.2	n.s.

Data presented as number (%) or mean ± standard deviation

*APACHE II* acute physiology and chronic health evaluation II, *Arg-to-Orn* Argininemia-to-ornithinemia, *SAPS II* simplified acute physiology score II, *SOFA* sequential organ failure assessment, vs versus

^a^Qui-square test

^b^Kaplan-Meier actuarial survival and log rank test

**Fig. 3:**
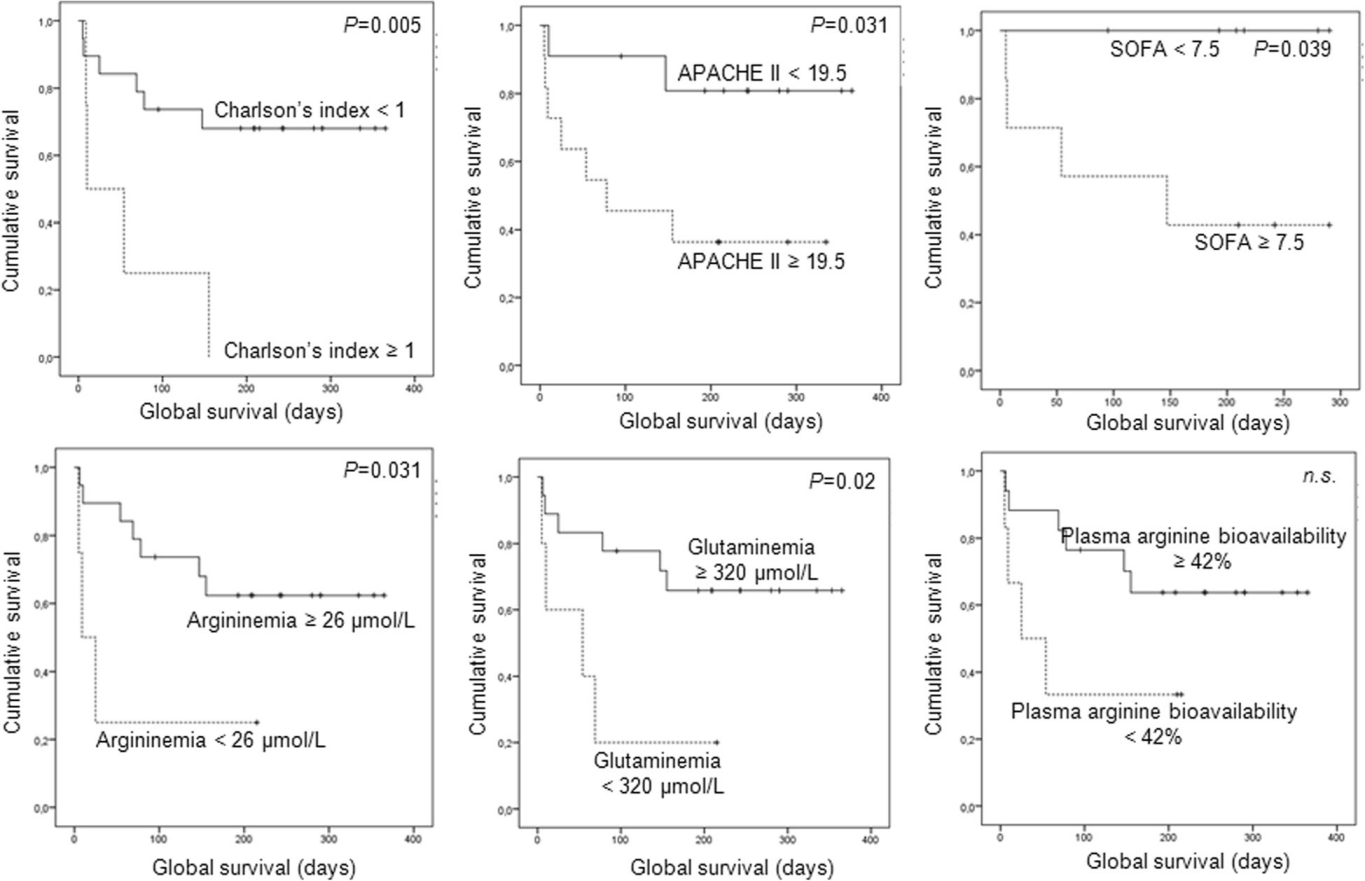
Actuarial survival curves in critical trauma patients (*n* = 23) admitted in the intensive care unit according Charlson’s index, APACHE II score, SOFA score, argininemia, glutaminemia and plasma arginine bioavailability at the moment of admission (Kaplan-Meyer curves and log rank test). *APACHE II* Acute Physiology and Chronic Health Evaluation II, *SAPS II* Simplified Acute Physiology Score II, *SOFA* Sequential Organ Failure Assessment

In univariate analysis, Charlson’s index superior or equal to one, APACHE II superior or equal to “19.5”, SOFA score superior or equal to “7.5”, argininemia lower than 26 μmol/L and glutaminemia inferior to 320 μmol/L were predictive factors of lower actuarial survival (Table 3; Fig. 3); none of those factors was significant in multivariate analysis.

Argininemia values inferior to 26 μmol/L, observed in 17.4 % of severe trauma patients, constituted significant predictive factors of in-hospital mortality [75 versus 15.8 %, *P* = 0.04; *odds* ratio = 4.7 (95 % CI 1.5–15.9); accuracy = 87 %; sensitivity = 57.1 %; specificity = 94.1 %; negative predictive value = 84.1 %; positive predictive value = 100 %] and of lower actuarial survival (63.5 ± 43.9 versus 256.1 ± 33.3 days, *P* = 0.031).

**Fig. 4:**
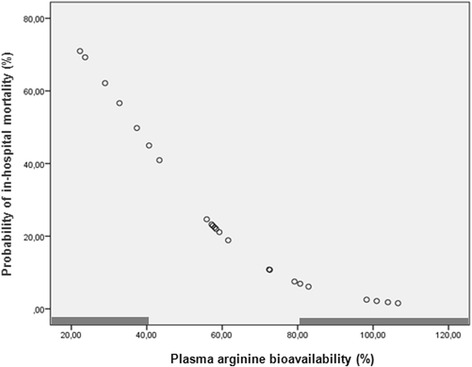
Probability of in-hospital mortality in critical trauma patients (*n* = 23) according the plasma arginine bioavailability (PAB) at the moment of the admission in the intensive care unit, calculated by the logistic regression model. Mortality was inversely and significantly related with PAB: 61.8 ± 8.8 % (95 % CI 50.8–72.7) when PAB inferior to 41 %, 21.3 ± 11.5 % (95 % CI 14.4–28.3) between 41 and 81 % and 2.8 ± 1.9 % (95 % CI 1.9–8.3) when PAB superior to 81 % (*P* = 0.0001)

PAB revealed a significant and high predictive capacity of in-hospital mortality [42.3 ± 14.7 % in cases of mortality versus 69.4 ± 25.1 % in remaining cases, *P* = 0.022; *auROC* = 79.4 ± 9.7 % (95 % CI 60.4–98.4), *P* = 0.032]. Probability of in-hospital mortality, calculated by the logistic regression model, was inversely and significantly related with PAB: 61.8 ± 8.8 % (95 % CI 50.8–72.7) when PAB inferior to 41 %, 21.3 ± 11.5 % (95 % CI 14.4–28.3) between 41 and 81 % and 2.8 ± 1.9 % (95 % CI 1.9–8.3) when PAB superior to 81 % (*P* = 0.0001) (Fig. 4).

PAB lower than 42 % at the admission, observed in 26.1 % of critical trauma patients, was associated with higher lactacidemia levels (3.68 ± 1.76 versus 1.95 ± 1.5 mmol/L, *P* = 0.033); higher in-hospital mortality [66.7 versus 11.8 %, *P* = 0.021; *odds* ratio = 5.7 (95 % CI 1.4–23.3), accuracy = 82.6 %; sensitivity = 66.7 %; specificity = 88.2 %; negative predictive value = 88.2 %; positive predictive value = 66.7 %]; and lower actuarial survival (87.2 ± 37.5 versus 261.4 ± 34.7 days, *n.s.*).

## Discussion

In the present study, mean arginine plasma levels of critical trauma patients at the admission on the ICU were lower than those of the historical healthy control individuals [29] and similar to those described in the literature for critically ill subjects [1, 32, 33].

Trauma is characterized by an arginine deficiency state associated with increased catabolism of this amino acid [5, 15], driven mainly by the marked upregulation of arginase in several tissues [4], proportional to the severity of the injury [34]; and further aggravated by the reduction of *de novo* synthesis and of dietary intake [4]. Trauma increases the expression of T-helper 2 lymphocytes, consequent to the activation of the hypothalamic-pituitary-adrenal axis and sympathoadrenal system, which cause impaired cell mediated immunity [5]. Furthermore, T-helper 2 cytokines increase the expression of arginase I in immature myeloid-derived suppressor cells causing arginine depletion, which further impairs T-lymphocyte function (increasing the risk of infections) and nitric oxide production [5].

In this series of trauma patients, argininemia at the ICU admission did not correlate significantly with severity and prognosis indexes. PAB lower than 42 % was associated with higher lactacidemia levels, considered markers of circulatory failure [32]. Arginase activity, estimated by the argininemia-to-ornithinemia ratio [8], was higher in severely traumatized patients at the ICU admission than in historical control individuals, as expected [5, 34]. Argininemia and PAB did not suffer significant variations during the first three days of ICU stay.

Unexpectedly, argininemia and PAB were not significantly related with the development of infectious complications. In fact, T-lymphocytes depended on arginine for proliferation and activation, zeta-chain peptide and T-cell receptor complex expression and development of memory [7]. The relatively high incidence of health care-associated infections (87 %) in this study may have prevented the observation of the effects of hipoargininemia.

In present study, PAB lower than 42 % revealed to be highly predictive of in-hospital mortality and argininemia values lower than 26 μmol/L were significantly associated with lower actuarial survival in severe trauma patients.

Previously, Gey A et al. [35] demonstrated a marked increase of granulocytic myeloid-derived suppressor cells in critically ill patients admitted in a medical ICU, which was inversely correlated with plasma arginine concentrations and overall survival. Other authors demonstrated, also, that high plasma levels asymmetric dimethylarginine (an endogenous inhibitor of NOS) and low arginine-to-asymmetric-dimethylarginine ratio constitute independent risk factors for organ failure and ICU mortality [13, 32].

Nevertheless, arginine supplementation in the critically ill patients remains controversial [4, 36, 37]. Analysis of its results has been hampered by the heterogeneity of the studied populations, differences in the arginine administration schedule (type of immune-modulating formula, timing and dosage), simultaneous provision of other immunonutrients and poor methodological quality of some studies [36].

High arginine-containing immune-modulating diets were recommended to be considered in severe trauma patients by the European Society of Clinical Nutrition and Metabolism [38] and by the Society of Critical Care Medicine/American Society of Enteral and Parenteral Nutrition [39]; on the contrary, routine arginine supplementation was discouraged in severe sepsis by both guidelines [38, 39]. Different responses to exogenous arginine in both arginine deficient states may be related with the predominant induction of arginase I after trauma while iNOS expression is increased in patients with sepsis [4, 5].

Putative deleterious effects of arginine administration in severe sepsis was attributed to the potential overproduction of nitric oxide by iNOS causing detrimental systemic vasodilatation with worsening of hemodynamic instability and peroxinitrite formation with cellular damage [40]. Nevertheless, adequate levels of nitric oxide seem to be necessary in sepsis to ensure organ perfusion [40–43]. According Gough MS et al. [8], in patients with severe sepsis, the ratio of arginine-to-dimethylarginine is reduced, proportionally to the severity of the illness, and predicts the outcome. Hirose T et al. [44] demonstrated that minimum values of argininemia were significantly lower in non-surviving than in surviving septic ICU patients. Recently, potential benefits of arginine monosupplementation during sepsis have been reanalyzed in experimental and clinical studies [24, 42].

Present series was characterized by the small number of studied patients and high severity scores. Nevertheless, hipoargininemia and low PAB represented pejorative prognostic factors. So, further research seems to be necessary to precisely identify patients that may benefit from arginine replacement, through biomarkers of the severity and type of arginine deficiency (such as argininemia, arginine bioavailability, arginase activity and nitric oxide metabolites). Additional dietary strategies to restore arginine plasma concentration can be studied, including the enteral supplementation of citrulline that, compared with arginine, may be associated with higher intestinal absorption, better gastro-intestinal tolerance, absence of hepatic uptake (without inducing urea synthesis) and minimization of the risk of excessive production of nitric oxide [45].

## Conclusion

In conclusion, present results confirm the high prevalence of arginine depletion in severe trauma patients and the relevance of argininemia (<26 μmol/L) and plasma arginine bioavailability (<42 %) as predictive factors of mortality in this context.

## Abbreviations

95 % CI: 95 % Confidence interval
APACHE II: Acute Physiology and Chronic Health Evaluation II
Arg-to-Orn: Argininemia-to-ornithinemia
auROC: Area under the “receiver operating characteristic curve”
ICU: Intensive care unit
n.s.: Statistically not significant
NOS: Nitric oxide synthase
r: Pearson’s correlation coefficient
SAPS II: Simplified Acute Physiology Score II
SD: Standard deviation
SOFA: Sequential Organ failure assessment
vs: Versus
